# Intersectionality of Cultural Identities in Health Psychology: Key Recommendations for Working With African-Caribbean Immigrant Women

**DOI:** 10.3389/fsoc.2019.00051

**Published:** 2019-07-23

**Authors:** Sandra Dixon

**Affiliations:** Faculty of Education, University of Lethbridge, Lethbridge, AB, Canada

**Keywords:** African-Caribbean immigrants, health psychology, cultural identity reconstruction, intersectionality, faith, social determinants of health, social justice

## Abstract

While the field of health psychology has progressed over the years, much work still needs to be done when considering immigrants' health care and well-being. Particularly, for African-Caribbean immigrants, the intersectionality of their cultural identities in the health care system require much attention moving forward. Therefore, this article is particularly relevant for Canada's multicultural society; it describes cultural identity reconstruction within health psychology as a common issue for diverse groups, particularly African-Caribbean immigrant women. The article speaks to the holistic worldview that is required in a paradigm shift which engages a pluralistic society that is Canada. The author presents a key cultural identity model and assessment tool that should be integrated into the health care system to ensure culturally-sensitive and inclusive care for immigrants, especially women. As is argued in the article, contemporary research, advocacy, and social movements speak to invoking alternative ways to complement the prevailing downstream approach to health psychology. In a growing multicultural society that should strive on honoring and respecting the pluralistic cultural worldviews of all people in the health care system, many immigrant women struggle to cope with the social determinants of health post-migration. Their concerns are often pushed to the margins of health care services, with several individuals relying on their faith for coping strategies. This article concludes with culturally-informed and socially-just recommendations for health care professionals working with immigrant populations, particularly African Caribbean immigrant women.

## Introduction

The Caribbean has one of the largest diasporas in the world in proportion to its growing population. As of 2016, there are 749,155 immigrants of Caribbean origin living in Canada, an increase of over 200,000, since 2011 (Statistics Canada, 2017a). Based on these numbers, women represent 399,345, which amount to 53% compared to 349,810 men, which equate to 47% (Statistics Canada, 2017a,b). Among this population, African-Caribbean immigrant women maintain unique historical and cultural experiences which must be considered by health care professionals working with this group. In the context of this paper, health care professionals will be the preferred term used, and include but are not limited to such persons as practitioners, clinicians, physicians, counselors, psychologists, nurses, and social workers. Historical roots of slavery, colonialism, and trauma, combined with difficult migration experiences, present unique challenges when navigating post-migration experiences (Model, 2008, pp. 125–126). Despite common exposure to troubling life events, the African-Caribbean community across the diasporas demonstrates low use of health care services and strong social stigmas, which create barriers for acknowledging mental health struggles (Sue and Lam, 2002, p. 409; Edge and MacKian, 2010, pp. 94, 105; McKenzie et al., 2011, p. 9). The intersectionality of salient aspects of African-Caribbean immigrant women's cultural identities like race, gender, and faith are often under-researched, especially within the Canadian context when addressing general health concerns and illnesses among this population (Dixon, 2015, p. 134; Mental Health Commission of Canada, 2016, p. 12). This is an area of grave concern since mental health is often stigmatized within this ethno-cultural community; such stigmatization may delay help-seeking due to fear and discrimination both from within and outside the African-Caribbean community (Edge and MacKian, 2010, pp. 95, 98; Mental Health Commission of Canada, 2016, p. 12). This gap in the health psychology literature requires further exploration to raise socio-cultural awareness and create health care resources that better serve this non-dominant group whom remain relatively invisible among those seeking/receiving help both in mainstream mental health services and associated research (Edge and MacKian, 2010, pp. 93, 105). In this paper, the term *non-dominant* is borrowed from Arthur and Collins's work and means groups who are systemically marginalized in society by being different from the dominant Anglo-Saxon, male, heterosexual culture (Arthur and Collins, 2010, p. 16).

Further, the African-Caribbean community also demonstrates unique strengths and resources which can be used to increase efficacy of mental health supports (Model, 2008, p. 142). It has been reported that about 79% of Caribbean individuals in Canada identify with a Christian faith, and of this number 54% are women while 46% are men (Statistics Canada, 2016). Many of these individuals consider religion to be a source of strength and guidance to help them cope with post-migration stressors (Dixon, 2015, p. 24). Such stressors include but are not limited to discrimination, racism, sexism, language barriers, unemployment, and educational plights. For many women of the African-Caribbean immigrant community in Canada, positive religious coping is found to act as a buffer against the above stressors and can be used to increase their resiliency and provide an avenue for comfort and growth (Dixon and Arthur, 2014, p. 95; Dixon, 2015, p. 158). Additionally, connections to family, heritage, culture, and faith communities are primary coping mechanisms for numerous African-Caribbean immigrant women and can be strengthened through health psychology research initiatives and mental health counseling practices to build on existing supports and values (McKenzie et al., 2011, pp. 6, 23; Sarafino et al., 2015, p. 80). Therefore, the argument can be made that the limited attention given to the cultural identity reconstruction of African-Caribbean immigrant women in the Canadian health psychology literature means that their experiences have not been fully explored (Dixon, 2015, pp. 213–215).

This conceptual analysis paper, therefore, is written to illustrate the multi-layered lived experiences of African-Caribbean immigrants post-migration and the intersectionality of their cultural identities within the profession of health psychology. Given that the experiences of African-Caribbean immigrant women across the diasporas are complex and multi-layered with significant socio-cultural intricacies, an in-depth discussion of their similarities/differences is beyond the scope of this paper. Acknowledging this limitation, the focus will be primarily on African-Caribbean immigrant women within the Canadian context whom are interculturally and intraculturally diverse and their narratives often excluded from mainstream health psychology discourse (Gagné and Veenstra, 2017, p. 376). First and foremost, a brief overview of health psychology will be provided to shed light on this rapidly developing field of study and the role that African-Caribbean immigrant women's post-migration cultural identity can play on their health. Attention will be given to the interplay of health and cultural identity reconstruction in the Canadian context. To define cultural identity reconstruction, an outline of the Afrocentric Model/Framework (Asante, 1988; Mazama, 2001) that is relevant to the lived experiences of African-Caribbean immigrants will be presented and critiqued. Parallelism will then be drawn between this model and its role in health psychology. Next, the paper will address salient dimensions of cultural identity, namely race, ethnicity, class, gender, socio-economic status, and religion/spirituality, and how these aspects of identities intersect to reconstruct new meanings for African-Caribbean immigrant women. Examination of these dimensions lead the author to advocate for a holistic critical health psychology model that considers the intersectionality of religion, race, and gender in the cultural identity reconstruction process for African-Caribbean immigrant women. This is followed by the role of social justice in health psychology for this population. The paper concludes with key practical recommendations for health care professionals working with immigrants, particularly African-Caribbean women.

## Overview of Health Psychology and Immigrants

Health psychology has evolved over the last 30 years and is considered one of the most burgeoning fields in contemporary academic psychology (Kaplan, 2009, p. 3; Sarafino et al., 2015, p. 11). In the context of this paper, *health psychology* refers to the study of psychological and behavioral processes in health, illness and healthcare (Blancarte et al., 1991, p. 189). Generally, the focus of a psychological approach to health relates to patients' psychological adjustments to, and management of, health problems (Sarafino et al., 2015, p. 11). The adjustment and management of health behaviors can be done through training and with the utilization of various psychological methods like biofeedback to control pain (Sarafino et al., 2015, p. 11). In contrast, the behavioral approach to health is primarily on presenting and modifying behaviors through educating and establishing reward/consequences (Elder et al., 1999, p. 275). *Health behaviors* are defined as actions taken by individuals that affect health or mortality; such actions may be intentional or unintentional and can promote or detract from the health of individuals or others impacted (Short and Mollborn, 2015, p. 78). Authors have critiqued the behavioral approach that it tends to place emphasis on individuals' behaviors, and thus neglects the broader health determinants, including income, migration status, and social environment (Dougherty, 1993, p. 111; Tengland, 2016, p. 24). This form of neglect can lead to victim blaming for diverse groups like African-Caribbean immigrants such as women who are often impacted by the above health determinants (Dougherty, 1993, p. 111; Tengland, 2016, p. 24).

Additionally, health and well-being consider a positive state of physical, mental and social functioning that vary over time along a continuum and not simply the absence of injury or disease (Sarafino et al., 2015, p. 2). The discipline of health psychology is making significant contributions to the prevention and treatment of chronic illness and focuses on the interface between biology, behavior, and social contexts (Taylor and Sirois, 2014, p. 3). Health psychology also overlaps with the related field of behavioral medicine, which emphasizes the interactions of behavior with biology and the environment, and the application of that knowledge to improve the health and well-being of individuals, families, communities, and populations (Kaplan, 2009, p. 3). From a broader perspective, both the Health Psychology Divisions in the Canadian Psychological Association and the American Psychological Association have gained momentum in recent years by conducting evidence-based and community-based research that explores the behavioral components and risk factors for disease prevention and health promotion in the North American health care system (Sarafino et al., 2015, p. 10).

Further, the Divisions of Health Psychology of the British Psychological Society and the European Health Psychology Society are also thriving (Kaplan, 2009, p. 3). Using a health psychology lens, the general goals of these Divisions are four-fold: (1) to promote and maintain health; (2) to prevent and treat illness; (3) to identify the causes and diagnostic correlates of health, illness, and related dysfunction; and (4) to analyse and improve health care systems and health policy (Sarafino et al., 2015, pp. 10-11). Arguably, these goals are relevant and require further examination to fully understand the precedents of health and health behaviors relative to the experiences of such groups like African-Caribbean immigrant women whose well-being plays a critical role in their overall functioning (Gurung, 2010, p. xv).

Within the Canadian context, health, and wellness are traditionally viewed through a biomedical model, which can be characterized as a disease management system that tends to provide a narrow and universal perspective on health and health care (Taylor and Sirois, 2014, p. 8; Sarafino et al., 2015, p. 12). A key critique of the biomedical model is that it utilizes a downstream approach to health in how individuals are symptomized, assessed, diagnosed, and treated (Meile, 2013, para: 1–10; Raphael, 2016, p. x). In other words, this approach refers to mostly individualistic factors such as genetics and medical conditions in health care and medical treatment planning (Merjudio, 2016, para: 3–5; National Collaborating Centre for Determinants of Health., n.d.). In contrast, many health care professionals are advocating for a biopsychosocial model that conceptualizes health care delivery from an *upstream approach* (Salami et al., 2017, p. 29). Such approach takes into consideration interventions aimed at examining the root causes of population health problems and illnesses such as social, behavioral, economic and environmental conditions (Australian Medical Association, 2007; Public Health Agency of Canada, 2016). Based on the above description, it is noteworthy to mention that cultural dimensions such as spirituality and religiosity are often excluded from the biopsychosocial model of health, which is a disadvantage for many individuals like African-Caribbean immigrant women whose faith keeps them mentally and physically grounded and plays a pivotal role in their cultural identity construction post-migration (Dixon, 2019; Dixon and Arthur, 2019). Used here, *faith* refers to subjective religious and spiritual beliefs and commitment to those beliefs (Koenig, 2005, p. 280).

To further expand, in reviewing the literature, spiritual and religious dimensions are often given limited space in the health psychology discourse, despite the key roles they play in mental health, physical functioning and well-being (Hansson et al., 2010; McKenzie et al., 2011). According to Koenig (2005, p. 143), most religious beliefs and practices help to maintain positive mental and social health, especially for many immigrant subgroups of African descent across the diasporas, like African-Caribbean women (Taylor et al., 2010; Dixon, 2015, p. 125; Dixon and Arthur, 2019). For these individuals, faith has helped to buffer against psychosocial and health issues like prenatal depression (Edge, 2008, p. 387; Edge and MacKian, 2010, p. 102) chronic health conditions such as cancer, AIDS, and diabetes (Nestel, 2012, p. 15; Goode et al., 2017, p. 27; Phillips et al., 2017, p. 54). It is well-documented that faith is closely tied to life satisfaction among many non-dominant cultural groups like people of African and Caribbean descent who are a demographically, religiously, linguistically, and culturally complex and diverse populations (Nestel, 2012, p. 15; Phillips et al., 2017, pp. 56-57). As such, Canada's health policy should consider adapting a holistic approach that values the spiritual and religious dimensions of many individuals, which are core aspects of their cultural identities.

Adopting a population health policy framework, Health Canada has highlighted 11 determinants of health: income and social status, employment and working conditions, education and literacy, physical environments, social support and coping skills, healthy behaviors, access to health services, biology and genetic endowment, gender, and culture (Government of Canada, 2018). Also, the Health Canada identification of social determinants of health includes culture but does not include race (Government of Canada, 2018). Growing evidence shows that race, racism and racialization are social determinants of health (Walker et al., 2016, pp. 366–371). Racialized groups are more likely to be in poorer health than non-racialized groups (Mikkonen and Raphael, 2010, pp. 32–33; Vang et al., 2017, p. 209). Given these racial factors, health care professionals should modify their intervention tools to ensure that the lived experiences of racialized groups including African-Caribbean women are considered.

Although the health determinants listed above reflect principles and practices of community development in health and health care interventions, the argument has been made that in the Canadian context immigrant women's experiences are not given much weight and should be included as a determinant of health (Hakim, 2001). This position is based on numerous studies that support the finding of a *healthy immigrant effect*, which concludes that immigrants' health is generally better than that of Canadian-born population pre- and post-migration; however, their health tends to decline after approximately 5–10 years in Canada (Ng, 2011, p. 1; Kennedy et al., 2015, p. 317; Vang et al., 2015, pp. 1–2). The decline of this foreign-born health advantage is likely credited to social exclusion, which in turn limits immigrants' access to social, cultural, and economic resources (Mikkonen and Raphael, 2010, pp. 32–33; Vang et al., 2017, p. 209). Therefore, the migration experience warrants a system of support and community care, which is emphasized by a holistic upstream approach to health (Hegar, 2016; Public Health Agency of Canada, 2016). With the realization that immigrants' health is significantly impacted by factors in their new environment, it is important to explore their cultural identity reconstruction (Dixon, 2015, 2019). Consideration should also be given to what mitigating variables and preventative measures health professionals should integrate into their models of care to help immigrants.

## Cultural Identity Reconstruction and African-Caribbean Immigrants

Over the years, much of the work on cultural identity has been explored by theorists in the United States (U.S.) who have studied the social constructs of race and related identities in the pan-ethnic collective of people ascribed the racial designation of *African-American* (Cross et al., 1991, pp. 319–320; Sullivan and Esmail, 2012). The concept *pan-ethnic* was coined by the U.S. sociologist Yen Le Espiritu, and refers to the collective bridging and solidarity among non-dominant groups who are racialized and labeled by the dominant culture (Espiritu, 2016). Although majority of the research on cultural identity has been completed in the U.S., there is sparsity of conceptual work that examines cultural identity reconstruction among African-Caribbean immigrants in the field of health psychology. The word *reconstruction* is used in this context because it reflects the lived experiences of African-Caribbean immigrants across the diasporas who undergo a process of reformation in their cultural identity post-migration (Hall, 1997a,b). This reconstruction process is complex and multidimensional, and is often ignored in health psychology discourse (Gore and Kothari, 2012, pp. 1, 14).

By definition, *cultural identity* is viewed as a multi-layered construct that represents “those aspects of our identities which arise from our ‘belonging' to distinctive ethic, racial, linguistic, religious, and above all, national cultures” (Hall, 1992, p. 273). Building on this definition, *cultural identity reconstruction* is adopted from the theoretical framework of social constructionism (Gergen, 1999, pp. 1–13). It reflects a part of the ongoing development of identity that is complex and fluid (Arthur and Collins, 2010, pp. 247–257; Dixon, 2015, 2019). This idea of reconstruction recognizes adaptation and negotiation within socio-cultural contexts where language plays a key role. It also refers to the ability of non-dominant immigrants to construct a salient identity based on a new geographical location and time (Hall, 2000; Dixon, 2015).

To offer a more in-depth conceptualization of cultural identity reconstruction that captures the lived experiences of African-Caribbean groups, reference is made to the work of Stuart Hall, a Jamaican-born cultural theorist and sociologist. In his famous essay, *Cultural Identity and Diaspora*, Hall proposed two theories of cultural identity. The first assumes that there is an essential cultural aspect to identity that is signified by either a common structure, common experience, or both (Hall, 1990, p. 223). In other words, one fully reconstructed identity replaces another superficial version of oneself (Bartholomew, 2012, pp. 24–25). The second position acknowledges that identities are constructed by partial fragments and are always in conflict with each other due to critical points of differences (Hall, 1990, p. 225). This view rejects the idea that identities are separate and distinct. Such perspective reflects the lived experiences of African-Caribbean women, many of whom have critical points of post-migration differences in their new socio-cultural and historical contexts (Hall and Du Gay, 1996; Hutchinson, 2014; Dixon, 2015, 2019). More so, through intersectionality of various cultural dimensions such as race, gender, and faith, African-Caribbean immigrant women's cultural identities merge and overlap to be multiple and/or fragmented identities rather than a singular identity (Bartholomew, 2012, p. 25; Dixon, 2015, p. 123). Undoubtedly, this author posits that Hall's above theorization of cultural identity re/construction is nuanced with complexity that requires further discussion and exploration, particularly in health psychology scholarship. For health care professionals who work with African-Caribbean individuals, this means that having a solid understanding of cultural identity models is important to incorporate into their work to increase cultural competency about diverse immigrant populations (Sue and Zane, 1987, pp. 37, 44; Dana, 1998, p. 3).

## Racial Identity and Cultural Identity Development Models

In recent years, the multicultural movement has given rise to consciousness in racial and cultural identity models that acknowledge non-dominant populations in North American contexts (Cross, 1978, pp. 16–18; Cross et al., 1991, pp. 319–320; Helms, 1995, p. 181; Berry, 2005, pp. 697–698; Sue and Sue, 2016, pp. 355–381). In social scientific inquiry, there is a lack of conceptual agreement across models about what constitutes cultural identity, particularly for non-dominant immigrant populations, like African-Caribbean immigrant women (Richardson et al., 2010, p. 227; Harvey et al., 2012, p. 22). According to Jackson and Meadows (1991, p. 72), conceptual frameworks may either hinder or facilitate individuals' understanding of the behaviors or experiences that occur in their lives and the lives of others. The above authors reason that change in conceptual frameworks provides new perspectives for understanding behaviors and experiences (Jackson and Meadows, 1991, p. 72). Therefore, key models of cultural identity reconstruction that relate to the experiences of non-dominant immigrants must both offer theoretical and empirical solutions for health behaviors and access to health care (Sarafino et al., 2015).

In critiquing the social science and psychology literature, one area of ambiguity is the distinction between racial identity and ethnic identity. *Racial identity* describes a collective group of individuals with defining features like skin, and eye color. In contrast, *ethnic identity* refers to a person's self-categorization and is part of one's overarching self-concept (Arthur and Collins, 2010, p. 14). While some theorists, use both concepts synonymously, other theorists view ethnicity as a subcategory of culture (Phinney, 1996, p. 918; Arthur and Collins, 2010, p. 14). In this paper, ethnicity is not viewed as a separate category but rather a sub-category of culture that embodies a broader concept. Based on the above descriptions, a majority of the existing cultural identity models tend to focus on the construct of race (Branch and Young, 2006; Sullivan and Esmail, 2012). According to Cross and colleagues, this tendency to overemphasize the construct of race promotes erroneous thinking in the literature (Cross et al., 1991, p. 319). Arguably, such skewed thinking could be of concern in the health psychology profession that already falls short in finding holistic interventions that fully address immigrants' health and wellness (Gurung, 2010, p. 529).

Consequently, the emergence of identity development models tend to be racially-oriented to address the issues of ethnic groups in the U.S., including African-Americans (Cross, 1971, pp. 13–27; Helms and Cook, 1999), Latino/Hispanics (Ruiz, 1990, p. 29; Casas and Pytluk, 1995, p. 155), and Asian Americans (Sue and Sue, 1971, p. 39). Additionally, African-Caribbean immigrants in Canada are often racialized in a similar manner, as the larger socio-cultural context influences the content, shape, and form of Black identity in terms of the intersectionality of race, class, socioeconomic status, and gender (Galabuzi, 2018, pp. 125–126). Research indicates that systemic racial issues impede the equal accessibility and distribution of resources to non-dominant groups, like immigrants (Galabuzi, 2018, pp. 139–140). These racial issues are well-structured to outweigh most other characteristics of ethnicity, such as culture, religion, and gender (Richardson et al., 2010, p. 227). Based on the understanding that the collective racial experiences may be similar for people of African descent across the diasporas, the models outlined below may be generalizable to the lived experiences of African-Caribbean immigrant women in the Canadian context, and possibly beyond. These models include: (1) The Cross Model (Cross, 1971, p. 13; Cross, 1995, p. 93), (2) The Helms Model or Minority Identity Development Model (Helms, 1995, p. 181), and (3) The Afrocentric Framework (Kambon and Baldwin, 1992; Belgrave et al., 2000, p. 386). However, given the conceptual framework of this paper, the Afrocentric model (Asante, 1988; Mazama, 2002, pp. 218–234) will be addressed due to its applicability to the lived experiences and worldviews of people of African descent across the diasporas. Below, this model will be addressed and critiqued for a better understanding in respect to immigrant populations.

## Theoretical Model/Framework for Cultural Identity Development

### The Afrocentric Model/Framework

Since the 1980s, a growing number of Black scholars have challenged the hegemony and normalization of Eurocentric theories of identity development (e.g., Asante, 1988; Mazama, 2001, p. 387; Bangura, 2012, p. 103). These Black scholars support a paradigm shift in theoretical frameworks, so that they consider the spiritual experiences of people of African descent across the diasporas. This paradigm shift has led to the development of the *Afrocentric model* (Randolph and Banks, 1993, pp. 204–214; Schiele, 1996, p. 284; Schiele, 1997a, pp. 21–39; Schiele, 1997b, p. 800; Mazama, 2002, pp. 218–234). According to Asante (1991), *Afrocentric* means “to study the world and its people, concepts, and history from an African worldview” (p. 271). This model proposes that Blacks across the diasporas share a common spiritual, historical and cultural legacy in terms of their identity (Asante, 2009).

Asante (1991, pp. 170–180) further argued that Afrocentrism places people of African descent and the interests of Africa at the center of Black people's approach to solving problems within a western context. For many Blacks, like African-Caribbean immigrants, Afrocentrism suggests a cultural pride that insists on being acknowledged and demands social justice, equity, and redress from a history of oppression (Este and Bernard, 2003, pp. 306–337). The above stance warrants consideration in health psychology discourse when it comes to health care treatment and policy-making that tends to exclude the lived experiences of non-dominant groups in Canada (Vang et al., 2015, p. 1).

As earlier mentioned, a paradigm shift is called for in the field of health psychology to develop a holistic framework that considers all aspects of one's cultural identity that might impact their ability to cope with social determinants of health (Purdy and Dupey, 2005, pp. 95–106; Gurung, 2010). This author argues that the Afrocentric model has areas of strengths that can be integrated into the health care treatment and promotion of various immigrant groups, including but not limited to African-Caribbean individuals (Randolph and Banks, 1993, pp. 204–214). Examined holistically, the Afrocentric model focuses on the values, assumptions, and beliefs of all people, such as African-Caribbean women, while acknowledging their innate spiritual nature (Randolph and Banks, 1993, pp. 204; Mazama, 2002, pp. 313). Drawing on Randolph and Bank's (1993, pp. 204–214) Afrocentric model, eight dimensions will be outlined and synthesized below for greater clarity and application to health psychology research. These dimensions are: (1) *spirituality*, (2) *communalism*, (3) *harmony and balance*, (4) *time as a social phenomenon*, (5) *affect sensitivity to emotional cues*, (6) *expressive communication and morality*, (7) *multidimensional perception and verve*, and (8) *negativity to positivity*.

First, *spirituality* represents a belief in a higher force or being. This conceptualization is subjective and transferrable across cultures. It also provides a framework for African-Caribbeans to view themselves, solve problems, and connect with each other. Secondly, *communalism* defines the idea that the group or collective outweighs the individual. It emphasizes the need for one to integrate one's goal with the greater community. The next dimension, *harmony and balance*, is the belief that human beings are an integral aspect of nature, as well as that all elements of life are connected and must be in balance. *Time as a social phenomenon* is another dimension that emphasizes that time is not its own entity, but it exists because of social interactions. *Affect sensitivity to emotional cues* signifies the awareness of one's feelings and the feelings of others as integral part of one's cognition, which is necessary for social adaption. *Expressive communication and orality* are considered key elements of the Afrocentric framework in how knowledge is conveyed. This dimension places value on oral over written expressions, as well as subjective and creative means of communications such as art and music. *Multidimensional perception and verve* represent multiple ways of learning, using all the senses, particularly rhythm and motion. *Negativity to positivity* is the final dimension of the Afrocentric framework. It illustrates one's ability to engage in health-conscious thought processes, to achieve positive outcomes out of negative situations. For many populations, particularly African-Caribbean women with a strong spiritual orientation, this means “making a way out of no way” (Harvey et al., 2012; Dixon, 2015, p. 34).

According to Mieder (2010), the above folk proverb is often used in the Black community to describe seeking God's help to cope with issues of marginalization and oppression. This reality was evident in Dixon's (2015) study that explored the experiences of a sub-group in Canada, Jamaican-Canadian immigrant women, who used their faith to help them make meaning of their post-migration cultural identity amid systemic issues like racism, discrimination, and feelings of being othered by the dominant culture within a multicultural society. These systemic factors are adverse effects of the immigrants' post-migration experience and should be addressed as part of health care intervention (Mitchell, 2017; Martis, 2018). In this vein, the Health Consciousness Scale (HCS) is a key assessment tool that could be modified and adapted into the health care system (Gould, 1990, p. 228). The HCS has been assessed for its validity and reliability and is said to mediate self-consciousness and health-promoting behaviors (Hassen and Kibret, 2015, p. 1). A recent study conducted at Jimma University, Jima Town, located in the western part of Ethiopia with a total sample of 110 teachers looked at the role of health-related behaviors and health consciousness in explaining psychological well-being. The outcome of this study revealed that participants who were health-conscious had positive health-related behaviors and psychological well-being, which influenced their optimal functioning and development during adulthood (Hassen and Kibret, 2015, p. 1).

There is a paucity of community initiatives in the Canadian context to address health-conscious behaviors through action on the social determinants of health (Gore and Kothari, 2012, pp. 2, 12). Addressing this paucity means that it might be useful for health care providers to make space in the health psychology profession to integrate culturally-sensitive and socially-informed assessment tools like the HCS into the health care process. The previous line of thinking can potentially help to promote safety and trust with immigrant groups as well as the argument for a critical health psychology approach for the purpose of working collaboratively across disciplines in the related area of social and health science (Hepworth, 2006, p. 331). The above approach will also contribute to fostering a holistic view of health that acknowledges the intersectionality of factors like race, gender and socioeconomic status (SES) that impacts racialized groups like African-Caribbean women.

## Intersecting Cultural Identities in Health Psychology

Immigration to Canada over the past decades has shaped the country, with an influx of immigrants adding to the nation's ethnic and cultural mosaic. Within Canada's multicultural society, immigrants have unique intersecting cultural identities that are complex and multi-faceted. Intersectionality theory proposes the idea that key dimensions of cultural identity including, but not limited to race, gender, and socioeconomic status interrelate with one another and can be indicators of chronic illness like hypertension and diabetes in avoidable ways (Gore and Kothari, 2012, pp. 2, 7). The concept of *intersectionality* was first introduced and developed by American civil rights advocate Kimberlé Crenshaw who called into question the racial discrimination against Black women who were filtered through a single categorical analysis in political and economic discourses (Crenshaw, 1989, pp. 139–140). In these spaces, the narratives were framed by men from the dominant Anglo-Saxon culture, and Black women were often marginalized and placed at the periphery of the narratives (Thomas and Crenshaw, 2004, p. 2).

According to Este et al. (2018, p. 28), intersectionality within the Canadian socio-cultural context can be characterized as a complex and multi-layered phenomenon based on socially constructed realities of oppression and domination. More so, key aspects of this phenomenon are multiple forms of inequality and disadvantages like gender, race, and other categories of differences in cultural identities that often compound themselves to create systemic barriers and perpetuate oppressed identities and social dislocations (Este et al., 2018, pp. 28–29). Such dislocations can influence societal structures like health care policies, health promotion and prevention, which inadvertently impact immigrants' health consciousness and psychological well-being (Gould, 1990, p. 128; Hassen and Kibret, 2015, p. 1).

Particularly for African-Caribbean immigrants, intersectionality takes on the form of the interplay between various forms of social injustices and systemic barriers to health and heath care inclusive of race, poverty, gender, and class (Bates et al., 2017, p. 2; Mitchell, 2017). Given the above perspectives, recognizing that immigrants are often referred to in the singular category despite their interplay of complex cultural identities need to be considered when working with diverse intersectional individuals like African-Caribbean immigrant women (Nestel, 2012, pp. 7–11). For example, the intersectionality of race, class, gender, and socio-economic status (SES) must be considered as social determinants amongst diverse immigrant groups in Canada (Gore and Kothari, 2012, pp. 1–2; McKenzie, 2015). Research indicates that systemic racial issues impede the equal accessibility and distribution of resources to non-dominant groups, like African-Caribbean immigrant women (Galabuzi, 2018, pp. 139–140). However, it is important to note that discrimination does not only just impact the care that people of African descent in Canada receive but also permeates every aspect of their holistic health (McKenzie, 2015; Mitchell, 2017). For example, many Black Canadians such as African-Caribbean women tend to be disproportionately affected by Human Immunodeficiency Virus infection and Acquired Immune Deficiency Syndrome (HIV/AIDS), mental health issues, heart disease, hypertension, sickle cell and stroke (Nestel, 2012, p. 15; McKenzie, 2015; Martis, 2018).

More specifically, African-Caribbean immigrants in Canada are reported to have a higher risk of acquiring diabetes than the general population, and complications from this illness are the leading causes of death in this sub-population (Jones, 2015, para: 6). Not surprisingly, recent Canadian report completed by the Wellesley Institute (Bates et al., 2017) on socio-demographic data and equity in health found that comprehensive and up-to-date research on discrimination in health care in Canada is not readily available. This is because compared to other places like the U.S. and the U.K., race-based information is not systematically collected across Canada (Department of Health, 2005; Bates et al., 2017, p. 17). Therefore, health care professionals' ongoing monitoring and understanding the sources of health disparities among African-Caribbean immigrant women in Canada could help to address upstream issues that increase the risk of illnesses that significantly impacts many members of this population (Bates et al., 2017, p. 5). To illustrate, research has shown that the intersectionality between race and socio-economic status contribute to social determinants of health and have been linked to chronic diseases such as cardiovascular disease, respiratory diseases, diabetes, and cancer (Yu and Raphael, 2004, pp. 366–368; Nestel, 2012, pp. 11–17).

Further, investigation on Canadian cities has found that people living in low income neighborhoods, particularly Indigenous groups, people of African descent, and women experience significantly higher rates of chronic diseases such as diabetes and earlier deaths than their wealthier White counterparts (Yu and Raphael, 2004, p. 366; Barnes and Snyder, 2012). With the knowledge that higher mortality rates are significantly impacted by race, gender, and income (Nestel, 2012, p. 19; Gagné and Veenstra, 2017, p. 371), a more holistic approach as previously suggested like the Afrocentric model in combination with the biopsychosocial approach need to be infused into population health in order to tackle upstream causes (Este and Bernard, 2006, pp. 9–12; Gore and Kothari, 2012, p. 1). Adopting a more holistic alternative to health care will allow for the equitable and justifiable exploration of health disparities and interrelations among ethnocultural groups to improve the well-being of all Canadians immigrants, irrespective of their cultural differences (Este and Bernard, 2006, pp. 9–12; Khan et al., 2017, p. 631).

Similarly to African-Caribbean immigrant women, African-American women experience mental health and racial disparities (Erving, 2011). For example, both groups show an association between internalized racism and depressive symptoms, with U.S.-born women in both groups exhibiting higher levels of internalized racism than African-Caribbean immigrant women (Mouzon and McLean, 2017, p. 1). This unique group difference is likely due to the fact that systems of racial stratification are common in a historically majority country such as the U.S., an entrenched pattern that stands in stark contrast to the experience of people of African descent residing in other areas across the African diaspora (Mouzon and McLean, 2017, p. 45). For instance, in many Caribbean countries like Jamaica and Trinidad, racial/ethnic minorities comprise the statistical majority of the population (Dixon, 2015; Changoor, 2018, pp. 17–18). It, therefore, follows that foreign-born African-Caribbean immigrants would experience an advantage, exhibiting the lowest levels of internalized racism relative to U.S.-born African-Americans and U.S.-born African-Caribbeans (Mouzon and McLean, 2017, p. 45).

Another key distinction between African-American and African-Caribbean women relates to help-seeking behaviors. According to Redmond et al. (2017, p. 312), both African-American and African-Caribbean groups report avoiding treatment for substance abuse due to wanting to solve their own problems, and not identifying a need for outside help. However, African-Americans were more likely to seek help from professionals than African-Caribbean women (Redmond et al., 2017, p. 312). The reluctance of help-seeking behaviors for African-Caribbean women in the U.S. align with the experiences of other women of African-Caribbean descent in the U.K. and Canada (Nestel, 2012; Office for National Statistics, 2018). Edge's (2013, p. 42) study on the intersectionality of ethnicity, gender, depression, spirituality, and implications for Black British Caribbean women's mental health in the U.K. highlighted an unexpectedly low rates of formally diagnosed perinatal depression in this sub-group, despite clinically significant morbidity. Given this outcome, maternal care is an area of grave concern for this population in the British health care system. Similarly, data collected by the U.S. Centers for Disease Control and Prevention (CDC) found that the maternal deaths for African-American women began to increase in the United States around 1990 (Roeder, 2019). According to CDC, African-American women are three to four times more likely to die during or after delivery than are White women (Roeder, 2019, para: 9). Further, African-American women are 243 percent more likely to die following birth than White women (Martin and Montagne, 2017, para: 10).

The above findings are staggering and signify grave concerns for women of African descent. However, such reluctance of help-seeking behaviors in both countries could likely be due to mistrust with a racialized medical system that tends to overdiagnose people of African descent with mental health issues like psychosis, and place less focus on other serious issues like maternity care for mothers of African heritage (Public Health Agency of Canada, 2016). Consequentially, the lack of maternal care for mothers of African descent has resulted in increased infant death rates for these individuals not only in the U.S. but other first world nations like Canada and the U.K. (Jarvis et al., 2005, p. 705; Public Health Agency of Canada, 2009; Nestel, 2012, p. 14; Office for National Statistics, 2018; Williams, 2018, para: 3–4). Disaggregating data by race reveals that higher rates of maternal and infant death among African-American women drive the U.S.'s mortality crisis (Novoa and Taylor, 2018, p. 2). This position is supported by Canada's national Maternity Experiences Survey (MES) that gathers data on women's experiences, perceptions, knowledge, and practices before conception and during pregnancy, birth and the early months of parenthood (Public Health Agency of Canada, 2009). According to this survey, immigrant mothers, First Nations, Inuit and Métis mothers are believed to be at increased risk for adverse pregnancy outcomes compared to their White counterparts (Public Health Agency of Canada, 2009, p. 11).

At this trajectory, it is important to acknowledge the health status of Canada's Indigenous groups whom like African-Caribbean immigrants are often othered within the health care system (Statistics Canada, 2018a). In this paper, the term *Indigenous* includes three categories: the Inuit, First Nations (Indians), and Métis people (First Nations Health Authority, 2018; Parrott, 2018, para: 3). Past report completed by the Health Council of Canada indicated that the key determinants of health on First Nations, Métis and Inuit peoples communities are influenced by several factors such as income and social status, lack of social support networks, unemployment and poor working conditions, gender, personal health practices, limited coping skills, and social displacement (Jamieson et al., 2005, pp. 20–30; Richmond and Ross, 2009, p. 403; Barnes and Snyder, 2012, para: 1–5). The argument has been made that Canada's health care system is quite color-coded, and as such it has failed to serve the best interests of racialized groups including African-Caribbean immigrants and Indigenous communities (Statistics Canada, 2018b).

With the understanding that different approaches can explain variances in health behaviors among non-dominant groups like Indigenous people and African-Caribbean populations, health care professionals need to be effective in addressing diverse beliefs, values, and attitudes (Gurung, 2010, p. 35). Further, health care professionals need to be culturally competent to explore diverse patients' understanding of health and sickness during the assessment process, to help them stay healthy and speed up the recovery process (Gurung, 2010, p. 35). As previously mentioned, historical forces, including experiences of slavery and colonialization relative to African-Caribbean and Indigenous populations have significant influence on health outcomes (Raphael, 2012, p. 49; First Nations Health Authority, 2018). Given the relevancy and ongoing psychological and generational traumatic impact of these historical events, health care professionals would do a disserve to patients if these factors in their social determinants of health are not considered. This awareness calls on health care providers to engage in social justice and advocacy work in the areas of health care and health promotion to ensure that the experiences of racialized and othered groups, including African-Caribbean women, are considered to ensure better health outcomes.

## Infusing Social Justice and Social Advocacy in Health Psychology

Social justice and social advocacy work remain fundamental to the practice of health psychology and should be infused into every aspect of the profession. Borrowing from Chang and Gnilka's (2010) definitions, *social justice* is the “belief in a just world that respects and protects human rights, while *social advocacy* describes the “act of arguing on behalf of an individual, group, idea, or issue in the pursuit of influencing outcomes” (p. 53). These descriptions reflect the key commitment of the United Nations to promote social progress, better living standards, and human rights for all people (United Nations System, 2016, para: 1). As such, at the 2007 World Summit for Social Development, this international organization declared February 20 of each year as the World Day of Social Justice (Montgomery, 2011, p. 3). Based on this global declaration, health care professionals within the scholarship of health psychology are called upon to develop culturally-sensitive and socially-informed health behavior practices, treatment plans, and interventions that are based on principles of equality and solidarity and that value and acknowledge the dignity of all people irrespective of age, race, ethnicity, social class, poverty, religion, gender, immigration status, sexual orientation, and language (Kenny and Romano, 2009, p. 23). With growing immigration comes the concerns of equitable treatment and distribution of societal benefits for all individuals, which is a strong consideration that must be recognized by health care providers who render services to immigrants cross-culturally in Canada, and beyond. For example, the ethno-cultural immigrant population in question—African-Caribbean women—have been reported to obtain lower wages despite their comparable or even higher educational achievements than members of the dominant group (Nestel, 2012, p. 9).

To demonstrate, past research on job earnings in Canada found that a native-born Canadian who belongs to a racialized group like people of African-Caribbean descent earned 18% less than a Canadian-born White man, whereas racialized Canadian-born women earned just 3% less compared to their White Canadian-born counterparts (Nestel, 2012, p. 9). Additionally, previous study conducted in Canada on the economic segregation and social marginalization of racialized groups has debunked the misguided societal assumption that wage discrepancies are results of educational deficits, since research outcome with immigrant population has found that the percentage of university-educated immigrants is more than five times that of native-born Canadians (Galabuzi, 2001, p. 71). Unquestionably, lack of employment and low wages contribute to poverty and limited access to health care services (Vang et al., 2017, p. 209). These inequities are forms of social injustices in Canada that affect the lived experiences of many African-Caribbean immigrants.

From a health psychology perspective, enhanced competencies in social justice advocacy are critical for health care professionals, since the traditional biomedical model falls short of efforts to overcome some of the social inequities that adversely impact persons from non-dominant groups (Sarafino et al., 2015, p. 7). Being that it is difficult to universally define social justice because of variations in personal experiences, health care professionals should become more culturally-minded by recognizing that there are culural identity differences in their work with diverse sub-groups including African-Caribbean women. Working from an upstream approach that considers all dimensions of a person's cultural identity, health care providers will be better able to deliver care that is physically and psychologically safe and secure for all cultural groups (Bell, 1997, p. 3). A key social justice model that health care providers might consider integrating into their work with diverse individuals is the Transdisciplinary Applied Social Justice model (TASJ).

Developed by Pratt-Clarke (2012, pp. 83–84), the TASJ model approaches complex social problems with a strategy that will increase the likelihood of successful social justice activism. Based on this notion, the TASJ model fits with the experiences of African-Caribbean immigrant women due to its Afrocentric theoretical grounding, which acknowledges the role of race and spirituality in the lived experiences of these individuals (Pratt-Clarke, 2012, pp. 95–96). This model is also transferrable to the lived experiences of other ethno-racial groups like Indigenous communities because of its common goal to understand how experiences are influenced by the operation of socio-cultural systems of power with the objective of identifying intervention strategies (Pratt-Clarke, 2010, Pratt-Clarke, 2012, pp. 38–39). This common goal has universal implications for the profession of health psychology that entails the promotion and maintenance of health through a commitment to praxis through social movements, which benefits all people in Canada's health care system as well as globally (Montgomery, 2011, p. 3; Pratt-Clarke, 2012, pp. 92, 94).

## Recommendations

The expanding field of health psychology in Canada has gained momentum in recent years with the influx of immigrants. As such, health care providers have become more intentional in making a conscious effort to recognize socio-cultural differences in health beliefs and behaviors across sub-populations like African-Caribbean and Indigenous communities (Sarafino et al., 2015, p. 18). In so doing, the governance of the health care system at the policy-making level has attempted to consider the social determinants of health like race, ethnicity, social support, income, employment, gender, and so forth that impact the physical and psychological functioning of diverse immigrants (Gagné and Veenstra, 2017, p. 371). Despite this strategic move in the right direction, significant evidence supports the deterioration of new immigrants' health status over time post-migration (Nestel, 2012, p. 10). Given this revelation, it is an area of serious concern for policy-makers, stakeholders, and health care providers to tackle for the betterment of health care promotion and accessibility for services across all cultures in Canada. Such a proactive approach to health care would help to address the growing plights of many people from non-dominant groups, including but not limited to individuals from African-Caribbean and Indigenous populations who struggle with chronic illnesses like diabetes, cancer, and hypertension (Goode et al., 2017, p. 19; Phillips et al., 2017, p. 52).

This paper by no means is intended to provide a comprehensive overview and assessment of the profession of health psychology and the Canadian health care system. Rather, this author makes the claim for a paradigm shift within health psychology discourse, which can potentially inform health and health promotion praxes. From this perspective, the author strives to stimulate further discussions about the topic in question, and drawing from the social science literature offers key recommendations for health care professionals to consider in their work with diverse populations like African-Caribbean immigrant women within Canada, and across the diasporas. Based on the premise of this conceptual paper, key recommendations are offered below for further reflections by the wider health care community, together with physicians, counselors, policy-makers, and stakeholders, all of whom might have some form of interactions with non-dominant groups on many different levels.

To start, it is critical for the above parties to be aware of the systemic issues that impact the lived experiences of all immigrants, including African-Caribbean immigrant women, such as race, gender, employment, SES and other discriminatory factors. More so, how the intersectionality of these factors shapes the cultural identity of immigrants and their lived experiences in Canada need to be given primary focus in health psychology literature and health care community contexts. Arguably, the social structure of the health care system could benefit from deconstructing its view on health and disease that is traditionally influenced by the biomedical model that adapts a downstream approach; rather, it could co-construct a population health upstream framework that is holistic in nature (Public Health Agency of Canada, 2016). This holistic framework will hold much value by drawing on the experiences of individuals in certain sub-cultures, like African-Caribbean immigrant women, whose narratives are often excluded from the policy-making process (Salami et al., 2017).

Building on the above point, Canada's health care system needs to take accountability for its role in designing a linear biomedical health care system that fails to consider socio-cultural multiple realities like the health beliefs, nutritional practices, religious/spiritual worldviews, and SES of many immigrant groups like African-Caribbean immigrant women (Taylor and Sirois, 2014; Sarafino et al., 2015, pp. 343, 348). To reiterate, for many people of African-Caribbean descent, faith plays a critical role in their ability to cope with post-migration life stressors and is predictive of changing health-related behaviors, like restraining from drugs and alcohol, that can inadvertently result in chronic diseases (Goode et al., 2017, p. 28). The health disparity in chronic illnesses like diabetes and hypertension that exist for many African-Caribbean immigrants is partly due to lack of health promotion strategies and accessible self-care management programs in these communities (Stoltzfus and Green, 2013, p. 16; Gagné and Veenstra, 2017, p. 371). It is, therefore, evidentiary to say that the religious and spiritual dimensions of African-Caribbean immigrant women should be given substantial consideration in their overall care and treatment planning.

Further, this author advocates for a health care delivery model that creates space for immigrants from non-dominant groups to play a proactive role in preventative health care measures. For example, it might be useful to obtain the valuable perspectives of immigrant communities in developing culturally-responsive assessment tools that infuse key dimensions of cultural identity such as diverse spiritual traditions and alternative healing practices to ensure best ethical decision making for all individuals (Canadian Psychological Association, 2000; Hays, 2016, pp. 127–160). This recommendation is influenced by the fact that there is a greater need for diverse research within the field of health psychology that draws from practice-based frameworks (PBFs) that are informed by social constructivist orientations and value multiple perspectives in the research process (Green, 2006, pp. 406–409).

Recurrent studies in the health psychology domain tend to rely primarily on evidence-based practices (EBPs) that are scientific in nature and often ignore the multi-dimensional aspects of socio-cultural and geo-historical contexts (Hays, 2016, pp. 227–228). Caution should be taken when critiquing EBPs within an ethnocentric framework that tends to be *culturally encapsulated* (Wrenn, 1962). This means that researchers are more likely to generalize interventions and treatments based on the dominant race and culture (Wrenn, 1962; Arthur and Collins, 2010, pp. 17–18). Therefore, it is important to consider the complexity and multi-layered experiences of immigrants such as African-Caribbean women and the key role of complementary and alternative measures in their overall health care (Gurung, 2010). For instance, faith and traditional healing practices can play a key role to improve the overall health and well-being of African-Caribbean immigrant women post-migration (Torri and Hornosty, 2017, p. xi).

Additionally, the argument can be made that due to systemic and structural barriers like micro and macro aggressions, there has been a lack of health care support and socio-political connection shown to pan-ethic groups like African-Caribbean immigrant women whose cultural differences are often othered and stigmatized by the dominant culture (Dixon, 2015, p. 27). Inadvertently, these differences frequently result in skewed research outcomes that are then generalized and misappropriated across non-dominant immigrant cultures (Razack, 2003, p. 338). To circumvent such outcomes, it would be prudent and strategic to involve insiders from within these immigrant communities to play an active role in the implementation and development of any initiatives and/or projects that impact the optimal health of their communities. For example, conducting action and community-based participatory research, which is dependent on a level of community involvement that may or may not entail an action or participatory component would be a step in the right direction for non-dominant groups, in order for researchers to demonstrate cultural inclusivity in ethical research practices (Padgett, 2016, p. 150).

More so, it might be important for policy-makers and stakeholders to re-evaluate the power differentials that exist in the health care system where the communities being served by health care professionals are excluded from the political discussions about policies that impact their well-being (Nestel, 2012; Bates et al., 2017; Office for National Statistics, 2018). Insiders from immigrant communities like African-Caribbean women are needed within the political arena to ensure that *all* cultural voices are being heard. Additionally, having an insider present at the health care policy-making level, will likely result in some degree of accountability from policy-makers. In alignment with this position, social justice activism that mobilizes change and influences hegemonic, structural, disciplinary, and interpersonal power domains are necessary to facilitate social movements for immigrants (Pratt-Clarke, 2012, p. 93). Taking a social justice stance in health care and health psychology provides a lens through which social problems can be analyzed and the examination of policies at the micro and macro levels can be upstreamed to benefit immigrants' cultural identity needs and health concerns (Pratt-Clarke, 2012, p. 83).

Lastly, this author posits that there is a call for health care providers to commit themselves to continuous professional development and training opportunities that stimulate growth around issues of cultural diversity (Nestel, 2012). Through critical reflections and reflexivity (Dixon and Chiang, in press), health care professionals can be better positioned to bridge some of the cultural gaps in Canadian society that tend to drive diverse communities apart due to their cultural differences instead of bind them together because of their unique commonalities (Satzewich and Liodakis, 2017, p. 122). More so, deeper self-explorations allow health care professionals to engage in socially-informed health care programs and practices that facilitate meaningful health change for immigrants. Such intentional engagement will in turn help to develop welcoming communities and social integration for immigrants like African-Caribbean women, which in turn will promote optimal health development for this population.

Research outcomes across various cultural contexts like the U.K., U.S., and Canada indicate that systemic racial issues impede the equal accessibility and distribution of resources to non-dominant groups, like African-Caribbean immigrant women (Edge, 2013; Galabuzi, 2018, pp. 139–140; Novoa and Taylor, 2018). As previously mentioned, there is a lack of extensive and current race-informed research completed across Canada's health care system in contrast to other countries like the U.S. and the U.K. (Bates et al., 2017 p. 17). In these countries, this form of race-based discriminatory investigation is given more attention, given its significant impact on community health, treatment planning, and policy-making initiatives for diverse populations (Department of Health, 2005). The author, therefore, argues that immense efforts should be placed into conducting similar nationwide research in Canada. This is because discrimination does not only just impact the care that non-dominant groups such as African-Caribbean immigrants in Canada receive, but it also permeates every aspect of their holistic health and well-being (Mitchell, 2017).

Based on the key recommendations outlined above, much work is needed in the implementation of these strategies at the various socio-political levels of government. By so doing, Canada's dominant culture will begin to celebrate and embrace diversity, and as such create inclusive spaces where the health needs of non-dominant groups such as African-Caribbean immigrant women are recognized and addressed in a more holistic, culturally-responsive, and culturally-sensitive manner.

## Author Contributions

The author confirms being the sole contributor of this work and has approved it for publication.

### Conflict of Interest Statement

The author declares that the research was conducted in the absence of any commercial or financial relationships that could be construed as a potential conflict of interest.
